# The Importance of Context: Uncovering Species- and Tissue-Specific Effects of Genetic Risk Variants for Type 2 Diabetes

**DOI:** 10.3389/fendo.2016.00112

**Published:** 2016-08-31

**Authors:** Soren K. Thomsen, Mark I. McCarthy, Anna L. Gloyn

**Affiliations:** ^1^Oxford Centre for Diabetes, Endocrinology & Metabolism, University of Oxford, Oxford, UK; ^2^Wellcome Trust Centre for Human Genetics, University of Oxford, Oxford, UK; ^3^Oxford NIHR Biomedical Research Centre, Churchill Hospital, Oxford, UK

**Keywords:** type 2 diabetes, genome-wide association study, genetic association, genetic mechanism, genomic annotation, gene regulation, epigenetics

Genome-wide association studies (GWAS) have been highly successful in identifying genetic variation associated with type 2 diabetes (T2D) risk and related quantitative traits (1–3). The vast majority of association signals are located in non-coding regions of the genome, influencing nearby genes through regulation of transcriptional, translational, or splicing activity (4). Due to the highly context-dependent nature of gene expression, the effects of many risk variants are restricted to specific cell types and produce more subtle effects than those observed in organism-wide (or “global”) knockouts. In addition, identification of the underlying causal genes and target tissues is often a major challenge, hindering translation into disease mechanisms. Recent studies have shown that the intersection of genetic data and genomic annotations can be used to produce a cellular atlas with which to understand the phenotypes of GWAS signals. Through the generation of directed hypotheses, this integrated framework has the potential to bridge the gap between association signals and disease biology.

## The Basis for Tissue Specificity

Across the human population, differences in complex traits, such as height and disease susceptibility, are influenced by the presence of single-nucleotide polymorphisms (SNPs). Some of these genetic variants modulate binding of transcription factors (TFs), which in turn drive differences in gene expression (5, 6). TF binding is also influenced by co-factors and chromatin state, which are highly dependent on cell type and developmental stage. To establish and maintain cellular identity, cell-type-specific TFs tend to bind in clusters, often referred to as cis-regulatory modules (7–9). Intriguingly, association signals for T2D have been found to show a significant overlap with islet-selective enhancer clusters (9, 10). Although other tissues have also been implicated in T2D susceptibility, this is consistent with physiological studies establishing islet dysfunction as a central mechanism of disease-associated variants (1, 11–13).

The overlap between T2D signals and enhancer clusters suggests that the effect of risk variants could be subject to the same tissue specificity observed at the level of regulatory activity (11). In other words, a motif-altering allele would only be expected to produce a molecular phenotype in those contexts (cell type or developmental stage) where the binding site has the potential to be occupied. In support of this notion, T2D risk variants were found to be enriched for nearby binding sites of the pioneer TF FOXA2 in islet and liver (14). T2D-association signals also show a significant overlap with SNPs affecting islet expression of regional transcripts, so-called cis-expression quantitative trait loci (cis-eQTL), most of which are not found to be eQTLs in other tissues (15). Together, these general observations outline some of the context-dependent effects that T2D risk variants can be subject to.

## Context Specificity of Causal Mechanisms for T2D Susceptibility

Recent studies have provided more specific evidence to support the notion that context specificity is a key aspect of GWAS causal mechanisms. The following cases collectively provide examples of mechanisms where studying the right tissue, species, and developmental stage proved critical to uncovering the relevant phenotypes.

At the *MTNR1B* locus, a convergence of evidence has pointed to effects of a non-coding T2D-association signal on the pancreatic β cell. Physiological studies have revealed a phenotype indicative of β-cell dysfunction in risk-variant carriers, with some evidence for additional effects on insulin action (12, 16, 17). Fine-mapping efforts identified a single likely causal variant that overlaps active islet and liver enhancers, and a cis-eQTL for *MTNR1B* in islets (14, 15, 18, 19). The risk allele, which increases islet *MTNR1B* expression, was predicted to create a NEUROD1 binding site and shown to selectively bind this key TF in human β cells. These results establish a likely causal mechanism for the non-coding risk allele, and illustrate how motif-altering alleles can generate highly tissue-specific effects. Surprisingly, exon re-sequencing of the *MTNR1B* gene has also shown coding loss-of-function (LOF) mutations to be associated with increased risk of T2D (20). The reason for the opposite directions of effect observed for coding and non-coding risk variants is unclear but may reflect differences between global and islet-specific roles of *MTNR1B*.

In the case of *PTF1A*, studying the right tissue proved necessary but not sufficient to elucidating the underlying mechanism for non-coding mutations in the region. Previous work had identified a group of patients suffering from unexplained isolated pancreatic agenesis, which includes neonatal diabetes as a clinical feature (21). To filter causal mutations from incidental variation, one study used pancreatic endoderm to define regulatory regions that are active during pancreatic development (22). Their strategy identified a distal enhancer that harbors mutations abolishing enhancer activity toward *PTF1A* (23). Coding LOF variants in *PTF1A* had previously implicated the gene in syndromic pancreatic agenesis, characterized by severe neurological features in affected individuals. The observation that the identified enhancer region is not active in any cell type other than pancreatic endoderm provides a plausible explanation for the absence of any cerebral defects (22). Remarkably, even adult pancreatic tissue did not show active chromatin marks in the region, highlighting that studying the right developmental stage was critical to the success of the approach.

The mechanisms underlying GWAS signals are sometimes studied using individuals that carry LOF mutations in positional candidate genes. The observed phenotypes will be a function of global effects across all the tissues where the gene is expressed, which may confound or mask the more context-dependent actions of regulatory risk alleles. At the *CDKN2A* locus, non-coding T2D signals have been robustly associated with measures of islet dysfunction, and a number of studies have established effects of *CDKN2A* on insulin secretion and cellular senescence in β-cells (12, 24, 25). By contrast, coding LOF mutations in *CDKN2A*, which are a cause of familial melanoma, were recently shown to result in a metabolic phenotype consistent with effects on both liver and β-cells (26). This discrepancy was proposed to arise from islet-specific TF binding of the enhancer region containing the T2D signals.

In animal studies, context-dependent knockouts can provide improved spatial and temporal resolution for targeting candidate causal genes. Even so, the disease relevance of the observed phenotypes is determined by the confidence with which the target tissue of the risk allele is known. One example is provided by an intronic T2D signal at the *TCF7L2* locus, which has been the focus of conflicting observations. Tissue-specific knockout studies have demonstrated primary roles of *TCF7L2* in a number of different tissues, including liver and islets, whereas the non-coding GWAS signal has been consistently associated with a relatively narrow insulin secretion defect. Genomic annotations provide a clue as to the underlying reason, with the risk variants being located in an islet-specific region of open chromatin (8, 10, 27). Furthermore, the region has chromatin marks indicative of regulatory activity in islets, but not in a wide range of other tissues (10). The annotations can, thus, be applied as a filter to exclude non-disease-relevant tissues, and guide efforts to study the effect of the risk allele in the most appropriate context.

Coding GWAS variants can also produce context-dependent mechanisms through the restricted expression of gene isoforms. A striking example of this is provided by a coding variant identified in the *TBC1D4* gene in a small founder population of Greenlandic Inuit (28). The risk allele, which produces a truncated transcript that results in nonsense-mediated decay, is positioned in an exon excluded from the short isoform of the transcript. Unlike the widely expressed short isoform, the long form is predominantly expressed in skeletal muscle (29). The decreased insulin sensitivity resulting from reduced expression of *TBC1D4* is, therefore, selectively imposed on muscle tissue. As a result, the risk variant has a different effect on fasting glucose from that observed in individuals carrying LOF mutations affecting both isoforms (28).

Similar to alternative spliceforms, gene homologs can contribute to concealing the primary effect of a GWAS signal in a specific context. At the *ADCY5* locus, T2D risk alleles have been linked to both decreased islet expression of the *ADCY5* gene and β-cell dysfunction, though the underlying molecular mechanism remains unclear (12, 15). Expression studies in rodents have shown *Adcy5* to be nearly undetectable compared with the closely related homolog *Adcy6* (30, 31). By contrast, human islets show roughly equal expression of these orthologous genes, hinting at a non-conserved function of *ADCY5* between the species (30, 32). It also highlights an underlying species-specificity that makes rodents less well suited as models for mechanistic studies.

## Limitations of Traditional Approaches

The examples above demonstrate that the molecular phenotypes of GWAS signals can be modulated by a multitude of context-dependent factors. In the case of non-coding variants, tissue- or developmentally restricted activity of the surrounding chromatin can limit effects on gene expression. For coding variants, alternative splicing or expression of homologs can mask a broader phenotype to produce context-dependent effects. These insights have important implications for how we design studies to translate genetic signals into molecular mechanisms.

**Figure 1:**
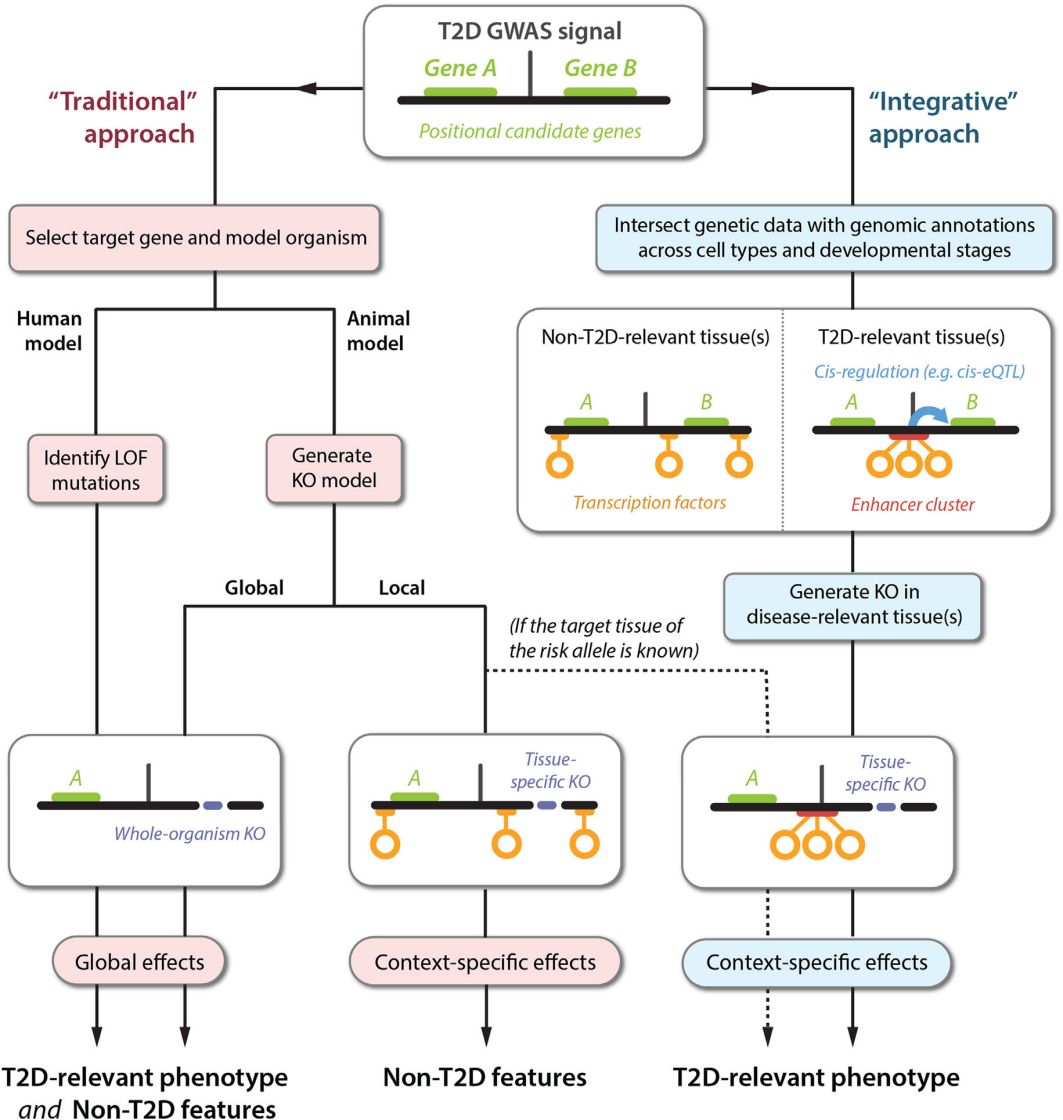
**Translating GWAS signals into disease mechanisms using traditional and integrative approaches**. The top panel shows a schematic representation of a GWAS locus with a non-coding association signal for T2D risk located near two genes, *A* and *B*. The left-hand side represents the traditional approach followed for elucidating the causal mechanisms leading to a T2D phenotype. This approach can produce a range of phenotypes that are difficult to translate into causal GWAS mechanisms, because the selected genes are often knocked out globally (bottom left panel), or in a tissue-specific context that is irrelevant to understanding the effects of the T2D susceptibility phenotype (bottom center panel). Sequencing approaches can also be used to identify gain-of-function mutations (not shown), which are subject to the same limitations. The right-hand side outlines an alternative approach using integration of emerging datasets to produce directed hypotheses. For optimal resolution, several types of genetic and genomic datasets can be integrated, including TF binding, enhancer regions (defined based on chromatin state), and cis-regulatory relationships (e.g., identified by cis-eQTL or chromatin-conformation capture studies). In this example, the GWAS signal is observed to disrupt an enhancer cluster with cis-regulatory activity toward gene *B*. Importantly, the disruption exclusively affects enhancer activity in disease-relevant tissues. This proposes a follow-up experiment for manipulation of gene *B* in a specific context (bottom right panel), producing a phenotype that is likely to be directly relevant to understanding the molecular basis of T2D susceptibility.

Traditionally, particular genes have been selected for follow-up studies based on a combination of known candidate-gene biology and proximity to the GWAS signal (Figure 1, *left*). For whole-organism gene knockouts, this could involve engineering of animal models or using genetic testing to identify individuals carrying LOF variants. As discussed, the relevance of these approaches for delineating disease-relevant mechanisms is limited by the potential for unspecific global phenotypes. Tissue-specific knockouts provide higher spatiotemporal resolution but require the target tissue(s) of the GWAS signal to be known. Human physiological associations can narrow down the list of likely relevant tissues, but these measures are often too crude to pinpoint specific cell types.

In principle, using animal models to target non-coding regions could produce more precise disease models, but this strategy is constrained by the low conservation at the level of regulatory architecture. In a study of the *Cdkn2a* locus in mice, targeted deletion of a 70 kb non-coding interval established enhancer activity toward nearby genes, but the relevance to humans has been questioned by subsequent findings (33, 34). The region encodes a long non-coding RNA that has no clear ortholog in rodents, highlighting the possibility of divergent cis-regulatory mechanisms. More generally, TF binding sites have been shown to diverge even faster than the underlying sequence itself (35). For two key liver TFs, the majority of binding events were shown to be species specific, while only 10–30% of hepatic enhancer clusters have corresponding rodent orthologs (36, 37). Although subsets of conserved clusters may aid in the prioritization of causal variants, these observations suggest that a different approach is required to delineate GWAS mechanisms (37, 38).

Even in those cases where animal models do provide targeted gene manipulation in an appropriate context (whether through tissue-specific knockout or transcriptional dysregulation), the resulting phenotypes may not be directly relevant for understanding human disease. Though rodent models continue to be an important tool for studying type 2 diabetes pathogenesis, it has become increasingly clear that murine pancreatic islets differ in a number of ways from their human counterparts (39–41). Certain monogenic forms of diabetes are, therefore, not well recapitulated in rodents, and molecular mechanisms elucidated in animal models should be interpreted with caution (42).

## Toward an Integrated Understanding of GWAS Signals

To successfully study disease-relevant phenotypes, experimental designs can be guided by the integration of genetic association data and genomic annotations (Figure 1, *right*). At the core of this framework is the overlaying of a static dataset – a list of variants linked to disease susceptibility and/or physiological traits – with layers of highly dynamic functional information that provide spatial and temporal dimensions. These layers encompass diverse datasets, and include information centered on single variants, such as histone marks and TF binding sites, and higher-order information that signifies relationships between distinct elements, such as chromatin interactions and cis-eQTL data.

Genomic annotations provide a cellular atlas with which to navigate and interpret genetic data in the context of specific cell types and developmental stages. For instance, if a set of likely causal variants has been identified from GWAS or fine-mapping studies, the tissue of action may be inferred from comparing chromatin states across cell types (8–11, 43–46). Conversely, if the target tissue is known from physiological associations, the causal mechanism can be pinpointed by overlaying with relevant functional annotations (14, 22, 47–51). This process generates a plethora of directed hypotheses that can be followed up with specific functional experiments. Increasingly, such studies are likely to be focused on differentiated cells derived from human stem cells, which can provide disease models and chromatin maps that are both functionally and developmentally relevant.

The broader applicability of this approach is, in part, determined by the tractability of individual loci. For regions with extensive linkage disequilibrium, the arising complexity can hinder experimental follow-up. Starting from a limited set of credible variants is, thus, essential. More generally, the value in taking an integrative approach is dictated by the extent to which genomic annotations for disease-relevant tissues have been made available (or can be obtained). The construction of a truly integrated framework is an incremental and monumental effort, facilitated by the Encyclopedia of DNA Elements (ENCODE) and the NIH Roadmap Epigenomics project, which together cover hundreds of tissues and epigenetic annotations. Since each dataset is merely a snapshot of a given cell type in a particular metabolic and developmental state, this on-going process will continue to produce an atlas with ever-finer spatiotemporal resolution. For many hard-to-obtain organs, such as pancreatic islets, power to detect relevant genomic features is still limiting.

Even so, chromatin landscapes for tissues relevant to T2D have begun to emerge in recent years, enabling biological inferences to be made. As we have seen, this has successfully uncovered tissue- and species-specific effects of T2D risk variants. The insights have also provided compelling evidence to demonstrate that context-dependent phenotypes are not the exception but, in fact, a fundamental aspect of GWAS biology. In the coming years, we need to build on this paradigm to accelerate the translation of genetic findings into molecular mechanisms.

## Author Contributions

ST, MM, and AG wrote, edited, and approved the manuscript.

## Conflict of Interest Statement

The authors declare that the research was conducted in the absence of any commercial or financial relationships that could be construed as a potential conflict of interest. The reviewer LL declared a past co-authorship with one of the authors MM to the handling Editor, who ensured that the process met the standards of a fair and objective review.
